# Prevalence and consequences of psoriasis in recent axial spondyloarthritis: an analysis of the DESIR cohort over 6 years

**DOI:** 10.1136/rmdopen-2021-001986

**Published:** 2022-01-28

**Authors:** Florian Lucasson, Pascal Richette, Krystel Aouad, Adeline Ryussen-Witrand, Daniel Wendling, Bruno Fautrel, Laure Gossec

**Affiliations:** 1 INSERM UMR-S 1136, Institut Pierre Louis d'Epidémiologie et de Santé Publique, Sorbonne Université, Paris, France; 2 Rheumatology Department, APHP, Université de Paris, Hôpital Lariboisière, Paris, France; 3 INSERM UMR1132 Bioscar, Université de Paris, Paris, France; 4 Rheumatology Department, Centre d'Investigation Clinique de Toulouse CIC 1436, Inserm, Paul Sabatier University, Toulouse University Hospital, Toulouse, France; 5 Department of Rheumatology, University Teaching Hospital, CHRU de Besançon, Besancon, France; 6 EA 4266 EPILAB, Université Bourgogne Franche-Comté, Besancon, France; 7 Pitié Salpêtrière Hospital, Rheumatology Department, APHP.Sorbonne Université, Paris, France

**Keywords:** spondylitis, ankylosing, patient reported outcome measures, biological therapy

## Abstract

**Objectives:**

The consequences of psoriasis associated to axial spondyloarthritis (axSpA) are unclear. The objectives were to determine the prevalence and the consequences of psoriasis in recent axSpA over 6 years of follow-up.

**Methods:**

The multicentric prospective cohort DESIR (NCT01648907) of adult patients with recent inflammatory back pain suggestive of axSpA was analysed over 6 years. Psoriasis was recorded at each visit and cumulative prevalence and incidence were calculated. Patients with vs without psoriasis at any time point were compared. Outcomes included disease activity (Ankylosing Spondylitis Disease Activity Score-C reactive protein (ASDAS-CRP), joint and enthesitis count, CRP), patient-reported outcomes for function (Health Assessment Questionnaire for axSpA, HAQ-AS) and quality of life, and treatment use over 6 years. Outcomes were compared through univariable and multivariable analyses, as well as linear mixed effect models.

**Results:**

In 589 patients, mean age 40.5±8.7 years, 45.8% men and baseline mean symptom duration 1.5±0.9 years, the cumulative prevalence of psoriasis increased from 16.8% (99/589) at baseline to 26.8% (158/589) at 6 years, leading to an incidence of 2.1/100 patient-years. Over 6 years of follow-up, patients with psoriasis developed more synovitis (p=0.008), and received more methotrexate (cumulative use, 25.5% vs 11.8%, p<0.001) and biological disease-modifying drugs (55.7% vs 38.5%, p<0.001). There were no significant consequences of psoriasis on other outcomes, including disease activity (ASDAS-CRP), functional capacity (HAQ-AS) and quality of life.

**Conclusion:**

Psoriasis is frequent in early axSpA. AxSpA patients with psoriasis had more swollen joints over time and received more biologics; they did not have worse outcomes related to axSpA in terms of activity and severity.

**Trial registration number:**

NCT01648907.

**Key messages**

**What is already known about this subject?**

- Psoriasis is frequent in established axial spondyloarthritis (axSpA). Some studies indicate that psoriasis may alter the course of axSpA.

**What does this study add?**

- Psoriasis was frequent (16.7%) in patients at the onset of axSpA.
- The prevalence of psoriasis increased regularly over 6 years of follow-up to reach a cumulative prevalence of 26.8%.
- Patients with axSpA and psoriasis had more swollen joints over 6 years and were more likely to be treated with methotrexate and biological disease modifying antirheumatic drugs than those without psoriasis.
- Disease activity and quality of life did not differ in patients with versus without psoriasis.

**How might this impact on clinical practice or further developments?**

- Patients with axSpA should be regularly monitored for psoriasis.
- Psoriasis modified the phenotype of axSpA with more peripheral involvement and more drug use, but without modifying the axial component of axSpA. These findings are important in the context of the current debate on psoriasis in axSpA.

## Introduction

Psoriasis, an immune-mediated skin disease,^1^ is a frequent extra-articular manifestation associated with axial spondyloarthritis (axSpA).^2^ The prevalence of psoriasis in established axSpA is estimated at 9.3% (95% CI 8.1% to 10.6%).^3^ However, as psoriasis in axSpA has mostly been assessed in cross-sectional studies,^3^ the time of onset of psoriasis is not well known, although there are data suggesting that psoriasis occurs early in the disease course.^4^

There is currently a debate on the role of psoriasis in axSpA. Differences in genetic risk factors between psoriasis and SpA have been highlighted: HLA-B27, the cardinal genetic variant associated with SpA, is not associated with psoriasis, whereas HLA-Cw6 confers the highest genetic risk of psoriasis.^5^ In addition, effective treatments differ between the two diseases. For example, the IL 12/23 inhibitor (ustekinumab) is not effective in axSpA,^6^ whereas it improves psoriasis and psoriatic arthritis (PsA),^7^ which suggests that underlying physiopathological and immunological mechanisms may differ between the two diseases. In view of these elements, some authors wonder whether axSpA associated with psoriasis corresponds to the same disease with different radioclinical presentation as axial PsA, or whether these are two different diseases.^5 8 9^ AxSpA patients seem to have more back pain at presentation and worse axial metrology compared with axial PsA patients.^8^ Moreover, axial radiographic involvement appears to be worse in axSpA than in axial PsA, with more bilateral sacroiliitis, complete sacroiliac joint ankylosis and bridging syndesmophytes in axSpA.^10^ More knowledge is needed on these two entities.

There is evidence suggesting that psoriasis worsens the rheumatic disease.^11 12^ It is known that psoriasis has a significant detrimental effect on quality of life, through its psychological and social impact.^13–15^ Furthermore, psoriasis patients are at higher risk of cardiovascular disease, in particular metabolic syndrome.^16^ In axSpA, patients with concomitant psoriasis appear to have a higher number of swollen joints, higher Bath Ankylosing Spondylitis Disease Activity Index (BASDAI) scores and to receive more treatments in comparison with non-psoriatic patients.^11^ The prognostic role of psoriasis in axSpA can be assessed by comparing patients with vs without psoriasis.

The objectives of this study were to determine the prevalence and incidence of psoriasis, to identify the profiles of patients who developed psoriasis and to assess the outcomes associated with psoriasis during the disease course of recent axSpA, including on clinical and structural outcomes and on treatment patterns. To this end, we analysed a large cohort of recent axSpA over 6 years of follow-up.

## Methods

### Study population and study design

The DESIRcohort (DEvenir des Spondylarthropathies Indifférenciés Récentes) has been previously reported; this longitudinal, prospective, multicentre French cohort included 708 adult patients (18–50 years old) with recent onset inflammatory back pain of more than 3 months and less than 3 years of duration, suggestive of early axSpA, between October 2007 and April 2010.^17^ Patients were followed up every 6 months the first 2 years and then annually with a planned follow-up of 20 years.^17^ For this analysis, data from the first 6 years were available and were analysed. At baseline, no patients were treated by biological disease-modifying antirheumatic drugs (bDMARDs). Each patient gave written informed consent.

### Variables of interest

#### Psoriasis

At each visit, presence or absence of psoriasis was recorded by the physicians as a dichotomous variable. Within the cohort protocol, there was no examination by a dermatologist; rheumatologists collected this diagnosis as reported by the patients or in the medical file, and whenever possible this information was confirmed by a medical report. Psoriasis was collected as a cumulative variable: once diagnosed, a patient was considered to have psoriasis and this variable was not collected at the later visits.

#### Baseline characteristics

Demographic and socioeconomic data were collected including age, sex, ethnicity. Clinical features were also collected such as body mass index, symptom duration, history of symptoms and family history. Disease activity was assessed through Ankylosing Spondylitis Disease Activity Score-C reactive protein (ASDAS-CRP), BASDAI, joint counts (28 swollen joint count, 53 tender joint count) and enthesitis (Maastricht Ankylosing Spondylitis Enthesis score, MASES, ranging 0–13).^18 19^ Health-related quality of life and functional capacity were assessed through patient-reported outcomes including the Health Assessment Questionnaire for axSpA (HAQ-AS), Bath Ankylosing Spondylitis Functional Index (BASFI), Ankylosing Spondylitis Quality of Life Scale (ASQoL) and Short Form 36 Health Survey Questionnaire (SF-36).^20 21^ Biological data (HLA B27 positivity, CRP) and imaging (spine and sacroiliac radiographs and MRI) were collected.

#### Consequences of psoriasis on recent axSpA

The primary analysis focused on two key outcomes: ASDAS-CRP and HAQ-AS, which were collected at each visit. Other outcomes included: BASDAI, BASFI, ASQoL, SF-36, days of work loss because of axSpA, joint and enthesitis count, CRP, and treatment use (non-steroidal anti-inflammatory drugs, conventional synthetic DMARDs and bDMARDs, analysed cumulatively as at least once during follow-up). Radiographic lesions were assessed through mSASSS (modified Stoke Ankylosing Spondylitis Spine Score which quantifies chronic spinal structural damage, ranging 0–72) and modified New York criteria^22^ on radiographs at baseline, 2 years and 5 years. Radiographs were independently scored for mSASSS by three trained readers (scores were averaged) blinded to chronological order, clinical characteristics and other imaging data.^23^

### Statistical analysis

#### Prevalence and incidence of psoriasis

The cumulative prevalence of psoriasis was calculated at each time point up to year 6 in the entire cohort and in patients with a follow-up visit at year 6. The incidence of psoriasis was estimated over 6 years of follow-up.

#### Baseline factors associated with psoriasis

Patients with a follow-up visit at year 6 were analysed. Quantitative variables were expressed as mean and SD and qualitative variables were expressed as numbers and percentages. Baseline factors collected were compared between patients with and without psoriasis over 6 years by univariable analysis (t-test or Mann-Whitney-Wilcoxon test for quantitative variables and χ^2^ test or Fisher’s exact test for qualitative variables, as applicable). A multivariable analysis using logistic regression was performed with covariates associated with psoriasis in the univariable analysis (p value threshold of 0.20), after checking for collinearity (correlation coefficient threshold of 0.80). The variables were also checked for clinical relevance by the investigators. HAQ-AS and BASFI on the one hand, and ASDAS-CRP and BASDAI on the other hand, were collinear. One variable from each of these groups (namely, BASFI and BASDAI) were entered in the next analyses.

#### Consequences of psoriasis on recent axSpA

Outcomes were compared between patients with vs without psoriasis using two methods:

- Univariable analysis and multivariable linear regression at year 6, including variables with p<0.20 and excluding collinear variables. The variables were also checked for clinical relevance by the investigators. This analysis was performed on the 589 patients present at year 6.
- Longitudinal models. In order to take into account the longitudinal nature of the data and to analyse the 708 patients, we performed generalised linear mixed-effects models with the patient as a random effect. Covariates associated with psoriasis at baseline were included in each model with a stepwise approach in order to select the best model based on the Akaike information criterion.^24^

There was no imputation of missing data.

## Results

**Figure 1:**
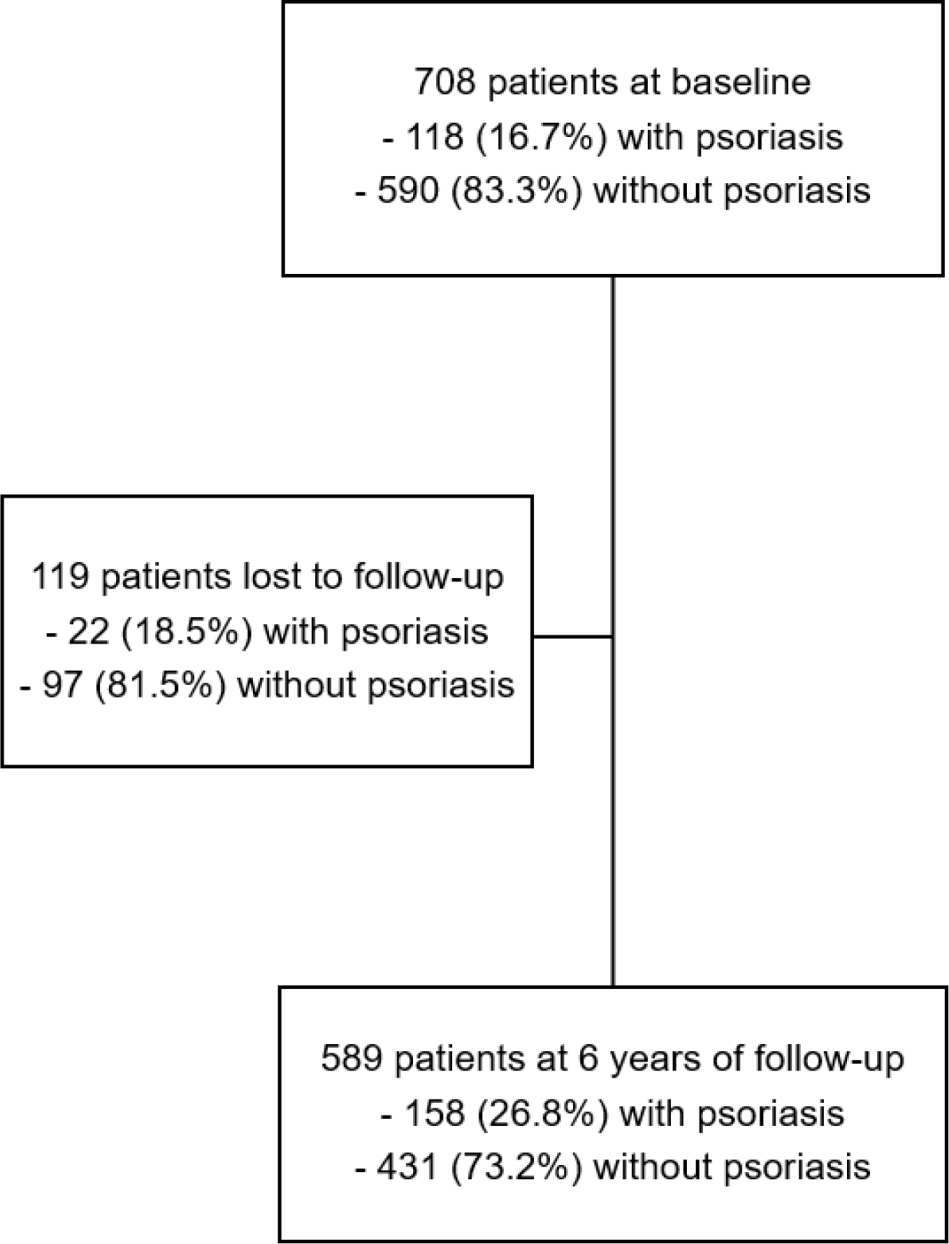
Flow chart.

Among the 708 patients included in the DESIR cohort at baseline, 92% fulfilled at least one set of classification criteria for SpA; 589 (83.2%) were followed up after 6 years (figure 1). Baseline characteristics of the 589 patients were globally similar to those of the overall cohort (data not shown) and included 270 (45.8%) men, mean age was 33.9 (SD 8.8) years, 213 (36.4%) were current smokers, mean symptom duration was 1.5 (SD 0.9) years and 357 (60.7%) patients carried the HLA B27 allele.

### Prevalence and incidence of psoriasis

**Figure 2:**
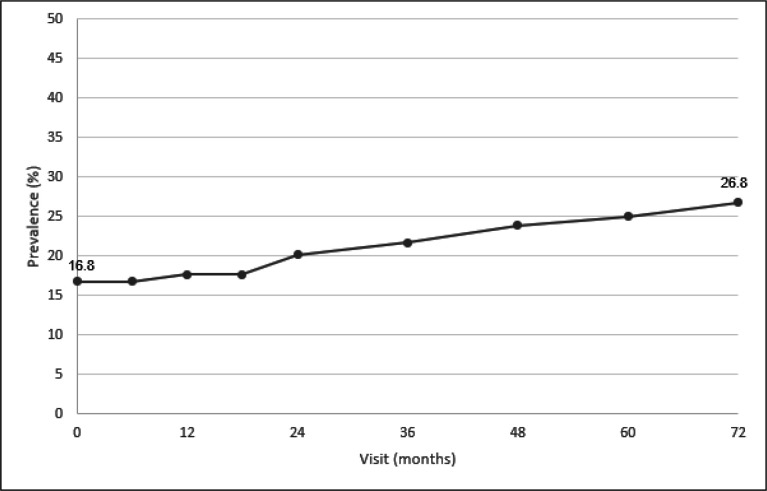
Cumulative prevalence of psoriasis in 589 axial SpA patients over 6 years of follow-up. SpA, spondyloarthritis. x-axis: visit (months), y-axis: cumulative prevalence of psoriasis (%).

At baseline, 118/708 and 99/589 patients had psoriasis leading to a prevalence of 16.7% and 16.8%, respectively (figures 1 and 2 and online supplemental table 1). At year 6, the cumulative prevalence increased to 26.8% (158/589), leading to an incidence of 2.1/100 patient-years. At the time of the DESIR assessments, no patients had psoriasis with a body surface area covered by psoriasis of more than 5%.

10.1136/rmdopen-2021-001986.supp1 Supplementary data

### Baseline factors associated with psoriasis at any time point

In multivariable analysis, a significant association with psoriasis was only found with Caucasian ethnicity (OR 4.08, 95% CI, CI 1.62 to 12.61), family history of psoriasis (OR 3.17, 95% CI 1.95 to 5.17) and a history or current baseline dactylitis (OR 2.59, 95% CI 1.36 to 4.91) (table 1).

**Table 1:** Baseline characteristics associated with cumulative psoriasis over 6 years of follow-up in 589 axSpA patients

	Psoriasis over 6 years of follow-up (N=158)	No psoriasis over 6 years of follow-up (N=431)	Multivariable analysis: OR (95% CI)
Age (years), mean (SD)	33.7 (8.6)	33.9 (8.9)	
Male gender, n (%)	67 (42.4)	203 (47.1)	
Symptom duration (years), mean (SD)	1.4 (0.8)	1.5 (0.9)	
Ethnicity (Caucasian), n (%)	151 (95.6)	382 (88.6)	**4.08 (1.62 to 12.61**)
Body mass index (kg/m²), mean (SD)	24.5 (4.7)	23.7 (3.8)	1.02 (0.97 to 1.08)
Past history or current symptoms of			
Peripheral arthritis, n (%)	59 (37.3)	102 (23.7)	1.26 (0.72 to 2.16)
Dactylitis, n (%)	35 (22.2)	43 (10.0)	**2.59 (1.36 to 4.91**)
Family history of psoriasis, n (%)	56 (35.4)	60 (13.9)	**3.17 (1.95 to 5.17**)
ASDAS-CRP, mean (SD)	2.8 (0.9)	2.6 (1.0)	
BASDAI, mean (SD) (0–100)	47.7 (19.3)	42.6 (20.4)	0.99 (0.98 to 1.01)
BASFI, mean (SD) (0–100)	35.7 (23.1)	27.2 (22.6)	1.01 (0.99 to 1.03)
Tender joint count, mean (SD) (0–53)	4.0 (6.8)	2.5 (4.9)	1.01 (0.97 to 1.06)
Swollen joint count, mean (SD) (0–28)	0.3 (1.2)	0.1 (0.7)	1.05 (0.83 to 1.40)
MASES enthesitis index, mean (SD)(0–13)	3.2 (3.5)	2.4 (2.8)	1.03 (0.95 to 1.13)
HAQ-AS, mean (SD)(0–3)	0.77 (0.52)	0.61 (0.51)	
ASQoL, mean (SD)(0–18)	10.2 (4.9)	8.7 (4.9)	1.0 4(0.97 to 1.12)
SF36, mean (SD)			
Physical Component Summary (PCS) (0–100)	38.6 (8.8)	40.9 (9.1)	1.00 (0.97 to 1.04)
Mental Component Summary (MCS) (0–100)	40.9 (11.5)	40.4 (10.9)	
MRI sacroiliitis (local reading), n (%)	54 (35.1)	156 (37.0)	
Modified New York radiographic criteria (local reading), yes, n (%)	21 (13.5)	80 (18.8)	0.80 (0.42 to 1.47)
Radiographic coxitis (local reading), n (%)	5 (3.2)	30 (7.0)	0.66 (0.20 to 1.82)
mSASSS (central reading), mean (SD)(0–72)	0.51 (1.9)	0.45 (1.4)	
HLA B27 positivity, n (%)	91 (57.6)	266 (62.0)	
CRP (mg/L), mean (SD)	8.5 (13.6)	8.0 (13.9)	0.99 (0.97 to 1.01)
csDMARDs, n (%)	28 (17.7)	54 (12.5)	1.19 (0.64 to 2.18)

Data were missing in <5% patients for symptom duration, body mass index, ASDAS-CRP, BASDAI, BASFI, tender joint count, swollen joint count, MASES enthesitis index, HAQ-AS, ASQoL, PCS, MCS, MRI sacroiliitis, Modified New York radiographic criteria, radiographic coxitis, HLA B27 and CRP. Data were missing in 42 (7.1%) patients for mSASSS. P values were <0.20 for all variables entered in the multivariable analysis; they were >0.05 for: body mass index, modified New York radiographic criteria, radiographic coxitis, csDMARDs. P values for HAQ-AS and ASDAS-CRP were <0.05 but they were not included in the multivariable analysis because of collinearity (HAQ-AS and BASFI on the one hand, and ASDAS-CRP and BASDAI on the other hand, were collinear. One variable from each of these groups (namely, BASFI and BASDAI) were entered in the next analyses.

Values in bold are significant values at p-value <0.05 in multivariable analysis

ASDAS-CRP, Ankylosing Spondylitis Disease Activity Score-C reactive protein; ASQoL, Ankylosing Spondylitis Quality of Life Scale; axSpA, axial spondyloarthritis; BASDAI, Bath Ankylosing Spondylitis Disease Activity Index; BASFI, Bath Ankylosing Spondylitis Functional Index; csDMARDs, conventional synthetic disease modifying anti-rheumatic drugs; HAQ-AS, Health Assessment Questionnaire for axSpA; MASES, Maastricht Ankylosing Spondylitis Enthesis Score; mSASSS, modified Stoke Ankylosing Spondylitis Spine Score; SF36, Short Form 36 Health Survey.

### Consequences of psoriasis on recent axSpA

#### Key outcomes

**Figure 3:**
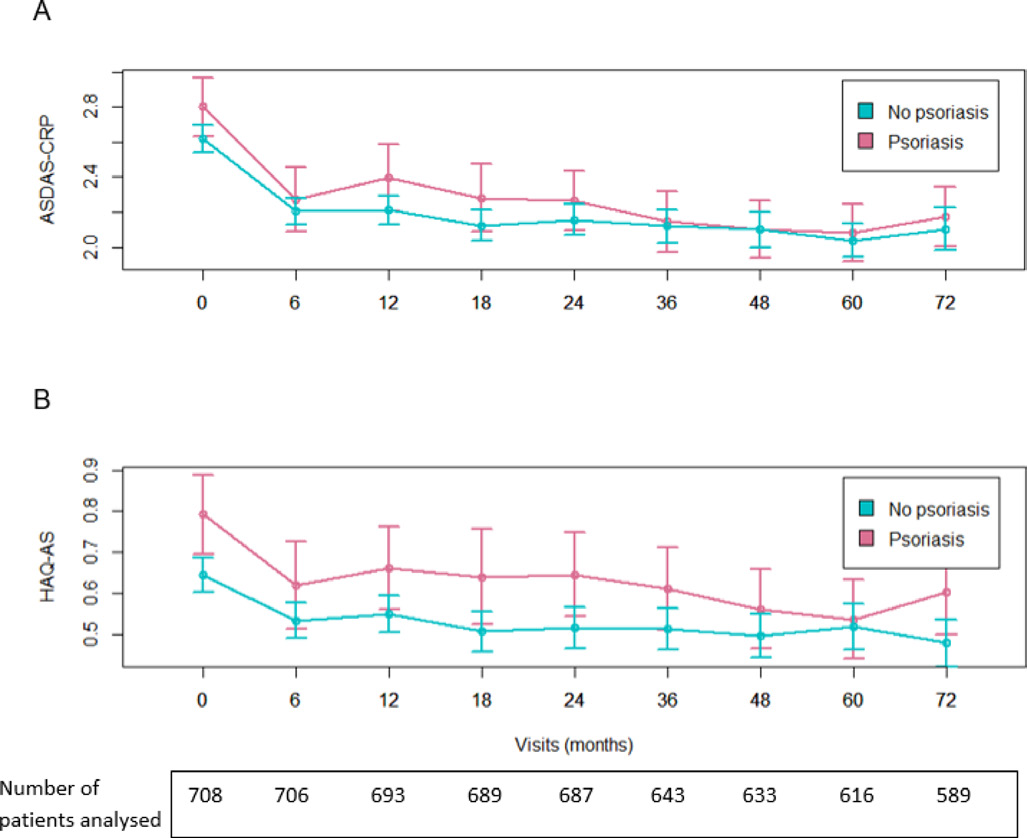
Evolution of ASDAS-CRP and HAQ-AS over time in patients with versus without psoriasis in axSpA patients. (A) ASDAS-CRP. (B) HAQ-AS. The x-axis is time in months, the y-axis is ASDAS-CRP in (A) and HAQ-AS in (B). The points represent mean values and the vertical bars represent the 95% CI. ASDAS-CRP, Ankylosing Spondylitis Disease Activity Score-C reactive protein; axSpA, axial spondyloarthritis; HAQ-AS, Health Assessment Questionnaire for axSpA.

ASDAS-CRP and HAQ-AS were similar in patients with vs without psoriasis. Figure 3 shows the comparisons over 6 years; differences were non-significant at 6 years of follow-up in both univariable and multivariable analyses (tables 2 and 3).

**Table 2:** Consequences of psoriasis on outcomes in axSpA patients in univariable analysis at 6 years (N=589) and over 6 years of follow-up (N=708)

	Psoriasis over 6 years of follow-up (N=158)	No psoriasis over 6 years of follow-up (N=431)	At 6 years: p value of univariable analysis (N=589)	Over 6 years: linear mixed-effects model: univariable effect estimate (95% CI) (N=708)
Key outcomes				
ASDAS-CRP, mean (SD)	2.2 (0.9)	2.1 (1.0)	0.36	0.03 (−0.07 to 0.13)
HAQ-AS, mean (SD) (0–3)	0.60 (0.56)	0.48 (0.49)	0.07	0.01 (−0.04 to 0.05)
Other outcomes				
BASDAI, mean (SD) (0–100)	36.3 (20.2)	32.8 (21.7)	0.09	0.55 (−1.61 to 2.71)
BASFI, mean (SD) (0–100)	25.9 (23.1)	21.8 (20.6)	0.13	−0.52 (−2.61 to 1.56)
ASQoL, mean (SD) (0–18)	7.2 (5.4)	6.4 (5.6)	0.10	−0.26 (−0.77 to 0.26)
SF36				
Physical Component Summary, mean (SD) (0–100)	42.7 (9.5)	43.5 (9.7)	0.37	0.03 (−0.91 to 0.97)
Mental Component Summary, mean (SD) (0–100)	43.3 (10.7)	44.8 (11.4)	0.07	−0.13 (−1.29 to 1.04)
Work loss (days per year), mean (SD)	20.9 (32.8)	21.2 (38.5)	0.70	
Tender joint count, mean (SD) (0–53)	3.4 (7.7)	2.2 (4.6)	0.11	0.20 (−0.66 to 1.07)
Swollen joint count, mean (SD) (0–28)	0.2 (1.3)	0.1 (0.3)	0.37	**0.08 (0.02 to 0.15**)
MASES enthesitis index, mean (SD) (0–13)	2.2 (3.1)	2.0 (3.0)	0.38	**0.31 (0.004 to 0.62**)
CRP (mg/L), mean (SD)	5.4 (8.5)	4.8 (6.7)	0.71	0.81 (−0.22 to 1.84)
mSASSS*, mean (SD)(0–72)	1.3 (4.7)	1.0 (3.0)	0.85	**0.23 (0.01 to 0.59**)
Change in mSASSS between baseline and year 5, mean (SD)	0.42 (2.0)	0.42 (1.7)	0.94	
Modified New York radiographic criteria during follow-up (local reading), n (%)	48 (30.4)	142 (32.9)	0.62	OR: 1.38 (0.57 to 3.35)
NSAIDs intake since last visit, n (%)	79 (63.7)	196 (66.4)	0.67	OR: 0.92 (0.63 to 1.35)
Current csDMARDs intake, n (%)	35 (44.3)	52 (34.9)	0.21	OR: 1.38 (0.70 to 2.74)
Methotrexate intake at least once during follow-up, n (%)	**37** (**25.5**)	**42** (**11.8**)	**<0.001** **OR: 2.57 (1.57; 4.21**)	OR: 2.05 (0.84 to 5.00)
bDMARDs intake at least once during follow-up, n (%)	**88** (**55.7**)	**164** (**38.5**)	**<0.001** **OR: 2.01 (1.39; 2.91**)	OR: 0.83 (0.38 to 1.81)

Effect estimates are beta unless otherwise indicated.

Values in bold are significant values at p-value <0.05 in univariable analyses at 6 years and over 6 years.

*Last assessment at year 5.

ASDAS-CRP, Ankylosing Spondylitis Disease Activity Score-C reactive protein; ASQoL, Ankylosing Spondylitis Quality of Life Scale; axSpA, axial spondyloarthritis; BASDAI, Bath Ankylosing Spondylitis Disease Activity Index; BASFI, Bath Ankylosing Spondylitis Functional Index; bDMARDs, biological DMARDs; csDMARDs, conventional synthetic disease modifying anti-rheumatic drugs; HAQ-AS, Health Assessment Questionnaire for axSpA; MASES, Maastricht Ankylosing Spondylitis Enthesis score; mSASSS, modified Stoke Ankylosing Spondylitis Spine Score; NSAIDs, non-steroidal anti-inflammatory drugs; SF-36, Short Form 36 Health Survey.

**Table 3:** Consequences of psoriasis on outcomes in axSpA patients in multivariable analysis at 6 years of follow-up (N=589) and over 6 years of follow-up (N=708)

	Multivariable analysis at 6 years of follow-up: effect estimate (95% CI) (N=589)	Over 6 years: linear mixed-effects model: multivariable effect estimate (95% CI) (N=708)
Key outcomes		
ASDAS-CRP	0.05 (−0.08 to 0.18)	0.01 (−0.05 to 0.07)
HAQ-AS(0–3)	0.05 (−0.05 to 0.14)	−0.003 (−0.04 to 0.03)
Other outcomes		
BASDAI (0–100)	−3.52 (−16.85 to 9.82)	0.76 (−0.09 to 1.62)
BASFI (0–100)	3.10 (−1.31 to 7.50)	−0.36 (−1.82 to 1.09)
ASQoL (0–18)	2.10 (−0.65 to 4.84)	0.07 (−0.19 to 0.33)
SF36		
Physical Component Summary (0–100)	0.69 (−5.97 to 7.35)	0.31 (−0.23 to 0.85)
Mental Component Summary (0–100)	−2.39 (−7.45 to 2.67)	0.14 (−0.64 to 0.92)
Work loss (days per year)	273.73 (−1070.26 to 1617.72)	
Tender joint count (0–53)	−3.78 (−10.92 to 3.37)	−0.10 (−0.83 to 0.64)
Swollen joint count (0–28)	−0.07 (−0.22 to 0.07)	**0.09 (0.08 to 0.16**)
MASES enthesitis index (0–13)	−0.10 (−0.87 to 0.68)	0.25 (−0.02 to 0.52)
CRP (mg/L)	3.50 (−9.60 to 16.61)	0.47 (−0.43 to 1.37)
mSASSS* (0–72)	−0.54 (−1.60 to 0.51)	**0.35 (0.03 to 0.68**)
Modified New York radiographic criteria during follow-up (local reading)	OR: 1.57 (0.22 to 11.71)	OR: 1.45 (0.56 to 3.73)
NSAIDs intake since last visit	OR: 1.14 (0.64 to 2.06)	OR: 0.95 (0.60 to 1.49)
Current csDMARDs intake	OR: 1.31 (0.45 to 3.74)	OR: 1.27 (0.60 to 2.66)
Methotrexate intake at least once during follow-up	**OR: 2.31 (1.09 to 4.97**)	OR: 2.38 (0.90 to 6.31)
bDMARDs intake at least once during follow-up	OR: 1.90 (0.99 to 3.69)	OR: 0.82 (0.34 to 1.99)

For each outcome of interest, the effect of psoriasis (yes/no) is reported after adjustment on variables associated with the outcome of interest in univariable tests (according to a p<0.20).

Values in bold are significant values at p-value <0.05 in multivariable analyses at 6 years and over 6 years.

*Last assessment at year 5.

ASDAS-CRP, Ankylosing Spondylitis Disease Activity Score-C reactive protein; ASQoL, Ankylosing Spondylitis Quality of Life Scale; axSpA, axial spondyloarthritis; BASDAI, Bath Ankylosing Spondylitis Disease Activity Index; BASFI, Bath Ankylosing Spondylitis Functional Index; bDMARDs, biological DMARDs; csDMARDs, conventional synthetic disease modifying anti-rheumatic drugs; HAQ-AS, Health Assessment Questionnaire for axSpA; MASES, Maastricht Ankylosing Spondylitis Enthesis score; mSASSS, modified Stoke Ankylosing Spondylitis Spine Score; NSAIDs, non-steroidal anti-inflammatory drugs; NSAIDs, non-steroidal anti-inflammatory drugs; SF36, Short Form 36 Health Survey.

#### Other outcomes

Patient-reported outcomes, included BASDAI, were similar between patients with vs without psoriasis (tables 2 and 3). AxSpA patients with psoriasis had more swollen joints and higher MASES enthesitis index over time (table 2) with a significant difference in multivariable analysis for swollen joints. Patients with psoriasis also had higher radiographic spinal damage (mSASSS) over time (tables 2 and 3); however, there was no statistical difference at the year 5 assessment and we found similar mean differences of 0.42 units over 5 years (table 2).

#### Treatments

Patients with psoriasis were more treated with methotrexate during the course of their axSpA as indicated in univariable and multivariable analyses, with ORs around 2, though this difference was not confirmed in linear mixed effect models (tables 2 and 3). Finally, patients with psoriasis were also more treated with bDMARDs as evidenced in univariable analysis (table 2). In multivariable analysis, the result was close to statistical significance (OR 1.90, 95% CI 0.99 to 3.69) but not confirmed in longitudinal models (table 3).

## Discussion

This study brings to light several important findings. First, psoriasis was frequent at the onset of axSpA and its prevalence increased regularly over time. Second, axSpA patients with psoriasis were more likely to develop synovitis and to be treated with methotrexate. Other outcomes including disease activity, functional capacity and patient-reported outcomes were similar in patients with and without psoriasis.

This study has strengths and limitations. In the DESIR cohort, patients were included if they had inflammatory back pain suggestive of axSpA, rather than a formal classification of axSpA. However, 92% patients fulfilled at least one set of classification criteria for SpA.^17^ Some patients were lost to follow-up and some had missing data over time which is frequent in longitudinal cohorts. However, the DESIR cohort is one of the largest cohorts of early axSpA^25–27^ and using linear mixed-effects models allows the analysis of every patient despite missing data. A key limitation is the way psoriasis was diagnosed and collected. Indeed, the diagnosis was recorded by the rheumatologist without a dermatologist assessment within the cohort procedures. Confirmation from the dermatologist was therefore not obtained to classify the patient as having psoriasis. From year 6 onwards, however, the information on the source of all new psoriasis diagnoses was collected and in fact all diagnoses at that time point were made by a dermatologist, which may provide some reassurance about the diagnoses in previous years. Moreover, the cumulative collection of psoriasis does not allow the assessment of psoriasis evolution over time. However, it is known that patients’ self-reporting of psoriasis is specific though may lack sensitivity (since patients tend to underestimate the prevalence of psoriasis).^28–30^ The number of outcomes analysed may increase the statistical alpha risk, however, we did not find links between psoriasis and most of the outcomes. Lastly, we applied different statistical analyses which were not always confirmatory. This could, however, be expected since univariable and multivariable analyses are cross-sectional whereas longitudinal models analyse data over time.

The prevalence of psoriasis at the onset of axSpA (here, in a population with a mean of 18 months of symptom duration) was particularly high in this cohort. Prevalence of psoriasis in early axSpA differs across studies, with values ranging from 4.4% to 13.3%.^4 25^ In our analysis, the prevalence of psoriasis increased regularly over time whereas a previous study indicated its early occurrence during the course of axSpA.^4^ These differences may be explained by different data collection methods. We believe our results show that regular monitoring of axSpA patients’ skin is of major importance.

Psoriasis was associated with three factors at the onset of axSpA in our analysis: Caucasian ethnicity, past or current dactylitis and family history of psoriasis. This is in line with previous research suggesting a higher rate of psoriasis in white compared with non-white ethnic groups.^31 32^ Furthermore, the diagnosis of psoriasis is more challenging on darker skin.^33^ Family history of psoriasis is a risk factor of psoriasis related to well-known genetic susceptibility, such as HLA Cw6.^16^ The more frequent peripheral involvement and dactylitis at the onset of disease in axSpA patients with psoriasis may suggest that these patients present manifestations of PsA. It would be interesting to know if these patients fulfil criteria for PsA; however, the CASPAR items for classification of PsA (including rheumatoid factor or psoriatic nail dystrophy) were not collected in the DESIR cohort.^34^ Thus, we could not confirm if the patients in our study would be considered as PsA according to CASPAR criteria. In our analysis, axSpA patients with psoriasis did not have some characteristics frequently associated with psoriasis, such as higher body mass index, cardiovascular comorbidities or anxiety and depression.^13^ These comorbidities are low in the DESIR population, and may appear later in the disease course. We can also hypothesise that axSpA patients with psoriasis present some differences from psoriasis patients alone.

In our analysis, we did not find that psoriasis worsened axSpA as the two key outcomes (ASDAS-CRP and HAQ-AS) were similar between patients with and without psoriasis. However, axSpA patients with psoriasis had more synovitis over time, suggesting again a phenotype close to peripheral PsA. This link between swollen joints and psoriasis has been suggested in previous studies, so our analysis provides additional arguments in this sense. Immunological and inflammatory mechanisms could explain this link between psoriasis and swollen joints. Indeed, since the interleukins modulating the inflammatory activity of psoriasis and axSpA are different, their combination could lead to a more extensive inflammatory process.^35^ As we evidenced a higher use of methotrexate and bDMARDs in axSpA patients with psoriasis over time, we can assume that these treatments were introduced to treat peripheral arthritis, especially since methotrexate is used in PsA and is particularly effective in peripheral joint disease, whereas it is not in axSpA.^7 36^ Nevertheless, we did not demonstrate that psoriasis worsens outcomes during the disease course of axSpA. Perhaps the effect of psoriasis was attenuated by the greater use of treatments previously mentioned. It should be noted that causality cannot be established with our analyses. A possible confounding factor that may increase the number of axSpA patients with psoriasis undergoing bDMARDs is the tumour necrosis factor-α (TNF-α) inhibitor-induced psoriasis phenomenon.^37^ In the DESIR cohort, 33 patients (4.7% of all patients and 18.3% of patients with psoriasis) were diagnosed with psoriasis after being treated with a TNF alpha inhibitor, though causality was not established. It is also noteworthy that the population in the DESIR cohort has a rather low/moderate disease activity, with a mean ASDAS-CRP activity score of 2.1. Thus, differences between patients are more difficult to highlight. The higher frequency of DMARDs use (methotrexate and bDMARDs) in axSpA patients with psoriasis may reflect a more active disease; it may also be related to the visual nature of psoriasis, leading either the patient or the physician to treat more actively the disease.

A higher mSASSS over time was observed in axSpA patients with psoriasis vs without. However, we observed a mean mSASSS difference of only 0.42 between baseline and year 5 in patients with and without psoriasis. Usually, radiographic progression is defined as an increase of ≥2 mSASSS units over 2 years.^38^ Thus, we believe the mSASSS differences are not clinically relevant. Of note, no differences were seen for radiographic sacroiliitis.

To conclude, our analysis revealed that psoriasis is a frequent extra-articular manifestation associated with axSpA which appears regularly over time, necessitating regular monitoring and multidisciplinary care with dermatologists. In the DESIR cohort, patients with psoriasis received more treatments. On the other hand, our analysis did not confirm the prognostic role of psoriasis in axSpA regarding disease activity, functional capacity, quality of life and radiographic lesions. These findings are important in the context of the current debate on psoriasis in axSpA.

## Data Availability

Data are available on reasonable request.
