# Supplementary material for: Prevalence and consequences of psoriasis in recent axial spondyloarthritis: an analysis of the DESIR cohort over 6 years
Source: RMD Open. 2022 Jan 28;8(1):e001986. doi: 10.1136/rmdopen-2021-001986 (PMC8804683; doi:10.1136/rmdopen-2021-001986)
Supplement: Supplementary data [file rmdopen-2021-001986supp001.pdf]

Supplementary Table 1. Cumulative prevalence of psoriasis in the DESIR cohort over 6 years of follow-up

	Inclusion	M6	M12	M18	M24	M36	M48	M60	M72
**Whole population (N=708)**									
Total number of patients with cumulative psoriasis in the entire cohort (lost to follow-up included)	118	118	125	125	141	150	163	169	180
Number of patients with cumulative psoriasis at each visit	118	118	125	122	139	141	152	156	158
Number of patients in the DESIR cohort with complete data	708	706	693	689	687	643	633	616	589
Prevalence of psoriasis (%)	16.7	16.7	18.0	17.7	20.2	21.9	24	25.4	26.8
**Population present at 6 years of follow-up (N=589)**									
Number of patients with cumulative psoriasis at each visit	99	99	104	104	119	128	141	147	158
Prevalence of psoriasis (%)	16.8	16.8	17.7	17.7	20.2	21.7	23.9	25.0	26.8

When taking into account patients lost to follow-up, there were overall 180 patients with psoriasis in the DESIR cohort over 6 years.

Prevalence of psoriasis was calculated at each visit in patients still present at 6 years (N=589) and in patients who completed each scheduled visit (ie, the whole population, N at baseline=708 then with decreasing numbers over time). The number of patients who completed the visit decreased over time so the prevalence of these two populations converges at 6 years.
